# Effects of the media conditioned by various macrophage subtypes derived from THP-1 cells on tunneling nanotube formation in pancreatic cancer cells

**DOI:** 10.1186/s12860-022-00428-3

**Published:** 2022-07-06

**Authors:** Chia-Wei Lee, Chia-Chen Kuo, Chi-Jung Liang, Huei-Jyuan Pan, Chia-Ning Shen, Chau-Hwang Lee

**Affiliations:** 1 Research Center for Applied Sciences, Academia Sinica, Taipei, 11529 Taiwan; 2 Genomics Research Center, Academia Sinica, Taipei, 11529 Taiwan; 3 Department of Biotechnology and Laboratory Science in Medicine, National Yang Ming Chiao Tung University, Taipei, 11221 Taiwan; 4 Institute of Biophotonics, National Yang Ming Chiao Tung University, Taipei, 11221 Taiwan

**Keywords:** Tunneling nanotube (TNT), Pancreatic cancer cell, Macrophage conditioned medium, Epithelial–mesenchymal transition, Mitochondrion transportation

## Abstract

**Background:**

Tunneling nanotubes (TNTs) are special membrane structures for intercellular communications. Vital cargoes (such as mitochondria) could be delivered from healthy cells to rescue damaged ones through TNTs. The TNTs could be utilized for the purpose of systematic delivery of therapeutic agents between cells. However, there are insufficient studies on the controlled enhancement of TNT formations. The purpose of this study is to understand how macrophages influence the TNT formation in cancer cells.

**Results:**

Here we compared the capabilities of inducing TNTs in human pancreatic cancer cells (PANC-1) of the media conditioned by M0, M1 and M2 macrophages derived from THP-1 cells. The M0 and M1 macrophage conditioned media promoted TNT formation. Using a focused ion beam to cut through a TNT, we observed tunnel-like structures inside dense cytoskeletons with scanning electron microscopy. The TNT formation correlated with raised motility, invasion, and epithelial–mesenchymal transition in the PANC-1 cells. Mitochondria and lysosomes were also found to be transported in the TNTs.

**Conclusions:**

These results suggest that TNT formation could be one of the responses to the immune stress in pancreatic cancer cells caused by M0 and M1 macrophages. This finding is valuable for the development of macrophage-targeting cancer therapy.

**Supplementary Information:**

The online version contains supplementary material available at 10.1186/s12860-022-00428-3.

## Background

Tunneling nanotubes (TNTs) are particular membrane structures for short- and long-distance (up to hundreds of micrometers) cell–cell communications. Two cells connected by a TNT can exchange various materials, including viruses [1], proteins [2], microRNAs [3], mitochondria [4], etc. The TNT formation is also thought to be one kind of stress response. For example, the PC12 cells treated by ultraviolet light formed TNTs with the untreated cells, and mitochondria were observed to be delivered from the healthy cells to the ultraviolet light-treated ones [4]. Moreover, as pancreatic cancer cells were treated by doxorubicin, the formation of TNTs were enhanced and the drug could be transferred between the cancer cells via the TNTs [5]. It seems that cells under various types of stresses can produce TNTs for delivering beneficial or detrimental components among nearby cells. It is straightforward to suspect that TNTs may also be involved in the complicated interactions between cancer cells in a tumor.

Macrophages are important constituents of the tumor microenvironment. The macrophages in a tumor microenvironment can be differentiated into various subtypes according to their T helper type 1 and T helper type 2 (TH1–TH2) polarizations [6]. The M1 (pro-inflammatory) macrophage can enhance drug sensitivities or decrease tumor activities [7]. On the contrary, the M2 (anti-inflammatory) macrophage tend to promote tumorigenesis or malignancy in some cancers [8, 9]. It is thus required to explore how these different types of macrophages influence the TNT formation in cancer cells.

In the present work, we studied the effects of conditioned media (CMs) of macrophage subtypes M0, M1, and M2, on the TNT formation in PANC-1 human pancreatic cancer cells. The M0 and M1 CMs made the PANC-1 cells produce more TNTs and increased the cell migration speeds. In contrast, the M2 CM was less effective on the TNT formation in the PANC-1 cells. We employed confocal microscopy and focused ion beam-scanning electron microscopy (FIB-SEM) to observe dense cytoskeletal networks inside a TNT. We also checked the correlation between the TNT formation and the epithelial–mesenchymal transition (EMT) of PANC-1 cells. In addition, bidirectional transportation of mitochondria between two cancer cells in the macrophage CM was observed.

## Results

### M0 and M1 macrophage CMs induced the formation of TNTs in PANC-1 cells

**Fig. 1:**
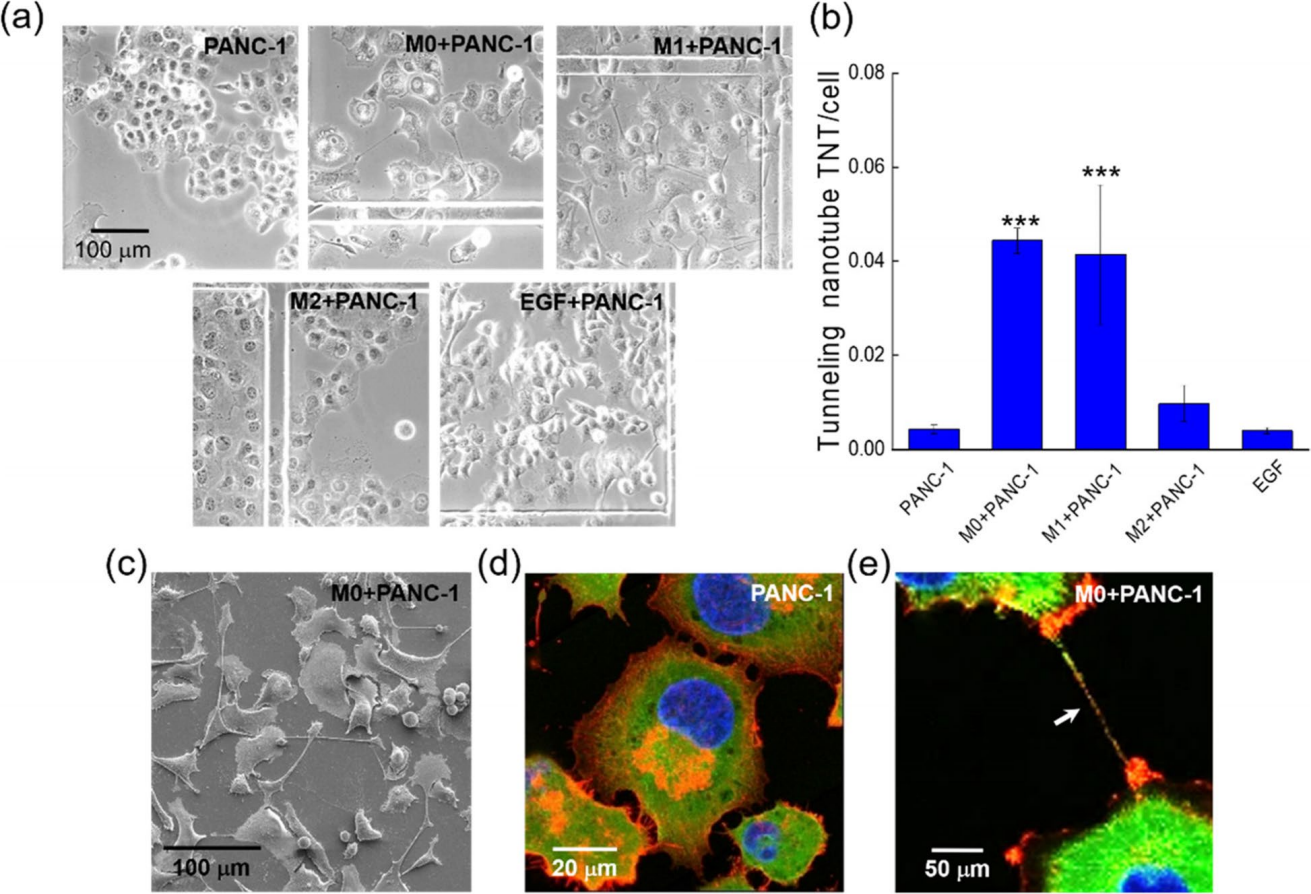
Macrophage conditioned media induce formation of TNTs in PANC-1 cells. **a** Typical morphology of PANC-1 cells in DMEM, macrophage CMs, and 200 ng/ml EGF respectively. **b** Average numbers of TNTs/cell in PANC-1 cells under the treatments of macrophage CMs and 200 ng/ml EGF. ***, *P* < 0.005 in comparison with the PACN-1 cells in DMEM (post hoc Tukey’s test). **c** Typical SEM image of PANC-1 cells cultured in M0 CM. **d**, **e** Confocal fluorescence images of cytoskeletons (green, microtubules; orange, F-actin) in PANC-1 cells in DMEM and M0 CM, respectively. The blue color represents DAPI-stained nuclei

In these experiments, the PANC-1 cells were originally cultured in DMEM for 24 h, and then we replaced DMEM with the macrophage CMs for another 48 h of culture. In the M0 and M1 CMs, the PANC-1 cells produced significantly more TNTs in comparison with those cultured in DMEM and the M2 CM, as shown in Fig. 1a. The PANC-1 cells cultured in DMEM formed compact colonies, while those in M0 CM and M1 CM were well separated and demonstrated TNT formation. The PANC-1 cells in M2 CM seemed to expand in size (compared with those in DMEM), but most of the cells were in contact with neighboring ones. In Fig. 1b we can see that the TNT formation probability was much higher in M0 CM and M1 CM. Figure 1c shows a typical SEM image of TNTs produced by PANC-1 cells cultured in M0 CM. Figures 1d and e show the redistribution of cytoskeletons (F-actin in orange and microtubule in green) near the membranes of PANC-1 cells. Without the treatment of M0 CM, the membrane protrusions in Fig. 1d contained mostly F-actin. In contrast, in M0 CM the TNT was filled with both F-actin and microtubules as indicated by the arrow in Fig. 1e. Therefore the cytoskeletons in TNTs might be different from those in membrane protrusions of the PANC-1 cells without the stimulations of macrophage CMs.

It was reported that epidermal growth factor (EGF) might play an important role in the TNT formation in rat breast cancer cells (MTLn3) [10, 11]. In our experiments, however, the addition of 200 ng/mL EGF into DMEM induced much less TNTs than the M0 and M1 CMs did (Fig. 1a and b). We also found very low concentrations of EGF in the macrophage CMs (Fig. S2(a) in the Supplementary Information). Therefore, we conjectured that the TNT formation in PANC-1 cells induced by the macrophage CMs might be irrelevant with EGF.

### Ultrastructures inside TNTs visualized with FIB-SEM

**Fig. 2:**
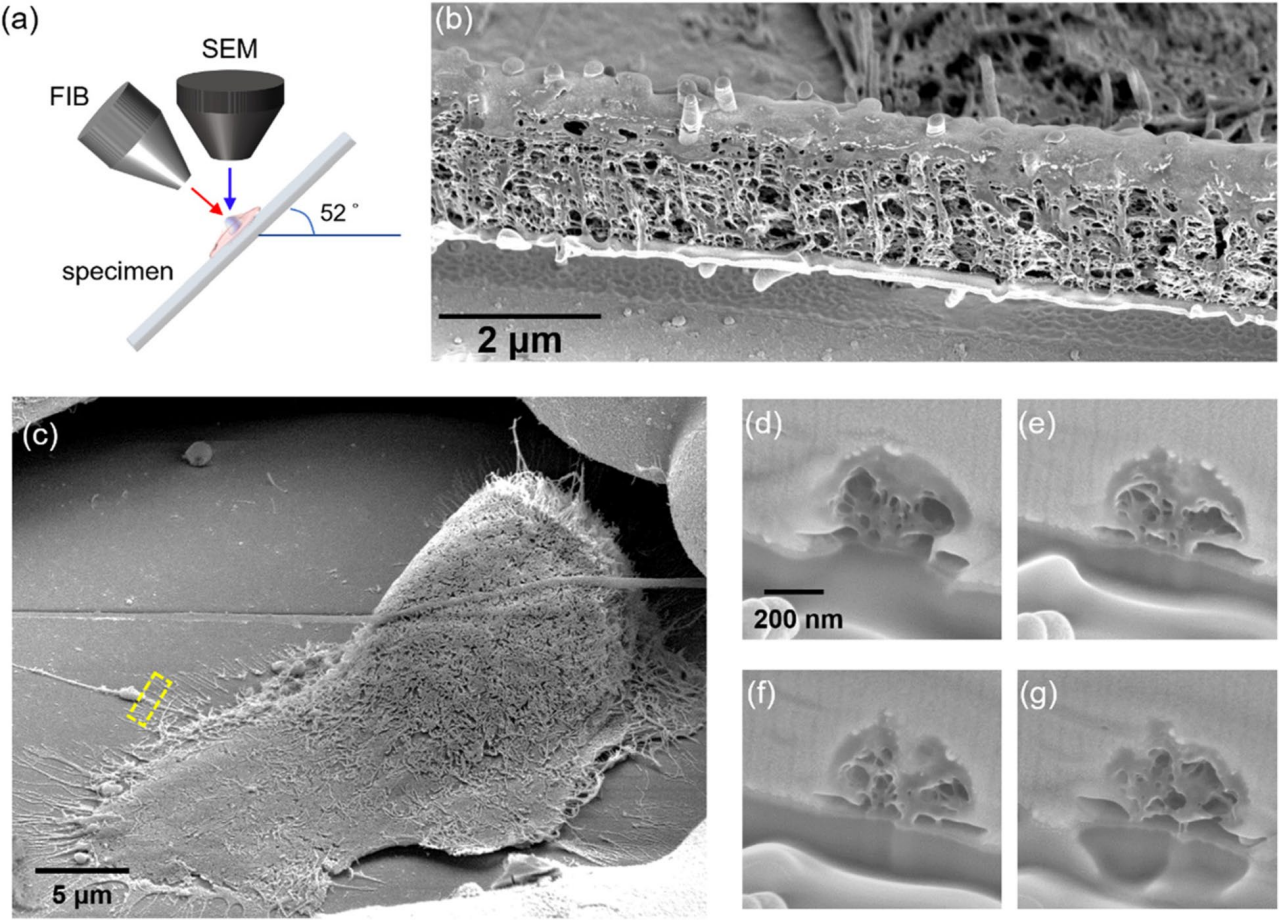
FIB-SEM images of the inner structures of TNTs. **a** Schematic of the FIB-SEM dual beam system. **b** SEM image of a longitudinally cut TNT between two PANC-1 cells cultured in M0 CM. **c** SEM image of a PANC-1 cell cultured in M0 CM. The region to be cut by FIB is marked by the yellow dashed rectangle. **d**–**g** Series of cross sections along the TNT in (**c**). Each section was separated from the previous one by ~ 500 nm

TNTs are not empty membrane tubes but filled with different cytoskeletons in different types of cells [12]. We observed that the TNTs between PANC-1 cells contained both actin filaments and microtubules, as shown in the fluorescence image in Fig. 1e. The TNT looked like an open-ended tube extending from the cell membranes, and both cytoskeletons inside the TNT were consecutive with those in the cell body. However, how the cytoskeletons are arranged in such a narrow space and how they are capable of delivering cargos were not resolvable by using optical microscopy. We therefore fixed the PANC-1 cells and dissected the TNTs by FIB, and then visualized the inner structures with SEM, as illustrated in Fig. 2a. The SEM image of a longitudinally cut TNT in Fig. 2b reveals that the TNT was filled with densely packed cytoskeletons. We also employed FIB-SEM to produce a series of cross-section images along one TNT (Fig. 2c–g). We observed some tunnel-like structures in the cross sections, surrounded by cytoskeletons. Because the diameters of the inner tunnels in the FIB-SEM images are around 100 nm or even smaller, the transportation of organelles such as mitochondria (diameters 500–1000 nm) could not be driven by passive flows of cytoplasms. The cargos might be transported through the TNT in a squeezed manner, similar to that reported in Ref. [13].

### TNT formation is correlated with enhanced cell motility and EMT

**Fig. 3:**
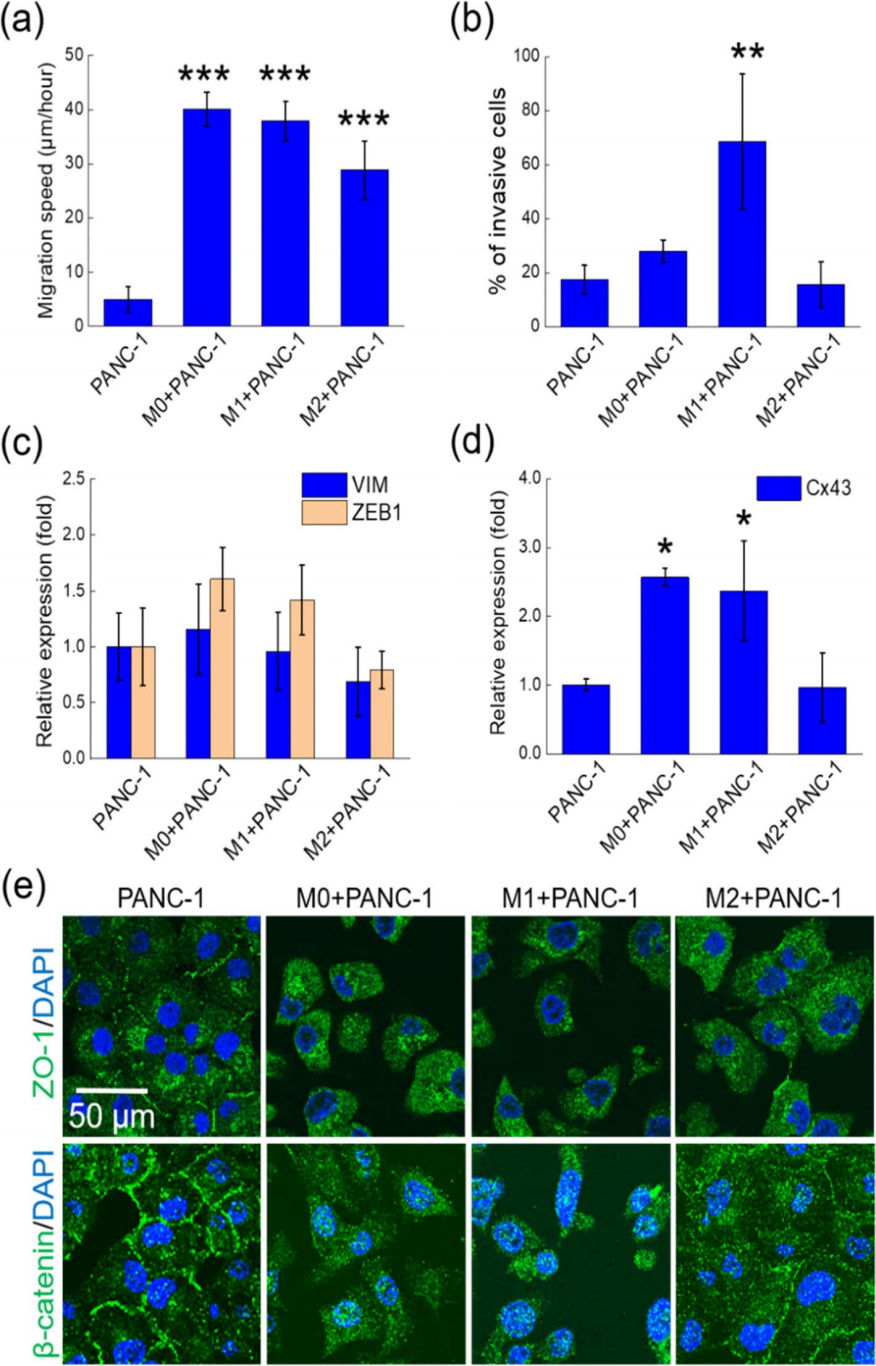
The TNT formation induced by macrophage CMs is correlated with enhanced cell motility and EMT. **a** Migration speeds of PANC-1 cells in DMEM and macrophage CMs. In each experiment, we measured the speeds of 45 cells. **b** Invasion in 24 h of PANC-1 cells in DMEM and macrophage CMs. **c** mRNA levels of vimentin (VIM) and Zeb-1 (ZEB1) in PANC-1 cells cultured in DMEM and macrophage CMs. For Zeb-1, the *P*-value between PANC-1 cells in DMEM and M0 CM is 0.17; while that between PANC-1 cells in DMEM and M1 CM is 0.34. **d** mRNA levels of connexin 43 (Cx43) in PANC-1 cells cultured in DMEM and macrophage CMs. All data were obtained from three independent experiments. ***, *P* < 0.005; **, *P* < 0.01; *, *P* < 0.05 (post hoc Tukey’s test) in comparison with the PANC-1 cells in DMEM. **e** Confocal fluorescence images of ZO-1 and β-catenin of PANC-1 cells in DMEM and macrophage CMs

In addition to the formation of TNTs, we also found that the M0, M1, and M2 CMs significantly increased the migration speed of PANC-1 cells (Fig. 3a), while only the M1 CM enhanced the cell invasiveness (Fig. 3b). We thus conjectured that the enhanced formation of TNTs might result from the dissociation of two cells originally in contact, known as the “cell dislodgement” TNT formation process [14], as shown in Fig. S3. It is straightforward to correlate the increase of cell migration speeds with EMT, which generally leads to higher cell motility and invasiveness [15, 16]. We used qPCR to check the mRNA levels of two EMT-relevant proteins, vimentin and Zeb-1 [17]. The mRNA level of Zeb-1 was increased by M0 and M1 CMs, although the difference was statistically insignificant (Fig. 3c). In addition, the mRNA level of connexin 43 was raised by M0 and M1 CMs but not M2 CM (Fig. 3d). Because connexin 43 is involved with the p-38 mediated cell migration [18], this result suggests that p-38 signaling pathway might be a part of the mechanisms in the migration acceleration caused by the M0 and M1 CMs in Fig. 3a. In contrast, the M2 CM did not cause the variation of vimentin, Zeb-1, and connexin 43.

We also monitored the spatial distributions of two cell–cell adhesion proteins, ZO-1 and β-catenin [15, 19], in the PANC-1 cells by using confocal fluorescence microscopy. The images in Fig. 3e show that ZO-1 and β-catenin moved from cell membranes into the cytoplasms when the cells were in M0 and M1 CMs. This process was consistent with EMT as well as the cell-dissociation behavior in Fig. 1a. The translocation of ZO-1 and β-catenin correlated with the TNT formation induced by M0 and M1 CMs in Fig. 1a. On the other hand, M2 CM did not induce the translocation of β-catenin from membranes into the cytoplasms. Perhaps this phenomenon could be relevant with the lower TNT formation in M2 CM.

### Mitochondria and lysosomes were delivered between PANC-1 cells through TNTs

**Fig. 4:**
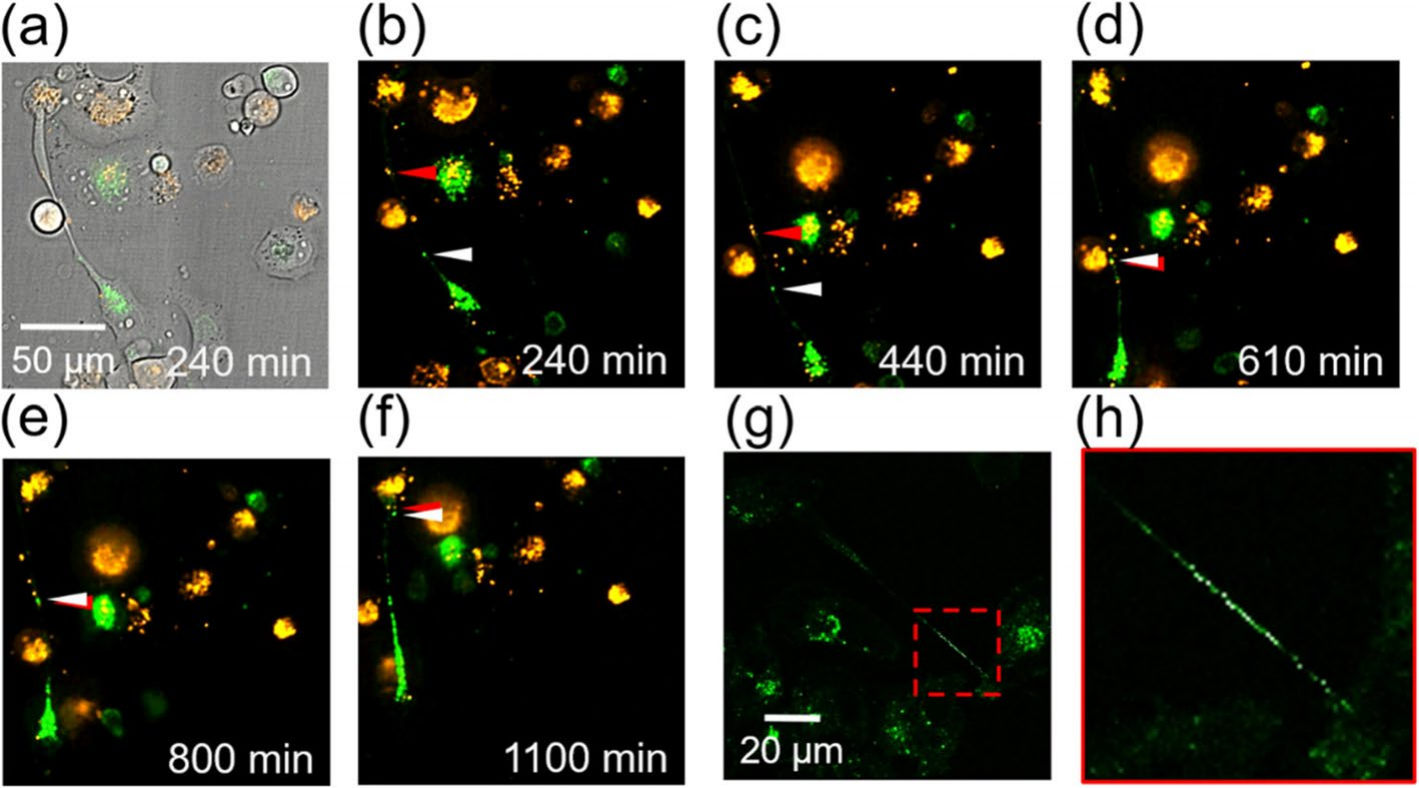
Mitochondria and lysosomes can be transported between PANC-1 cells in M0 CM connected by a TNT. **a** Overlapped bright-field and fluorescence images showing two PANC-1 cells connected by a TNT. **b**–**f** Time-lapse confocal fluorescence images of mitochondrial trafficking within the TNT in (**a**). The two PANC-1 cells were sub-cultured and labeled with MitoTracker™ Green FM and MitoTracker™ Orange CMTMR, individually. Two mitochondria from each cell moved forward in opposite directions, collided, and then moved together into the upper cell. **g** Fluorescence image of lysosomes in PANC-1 cells. The lysosomes were labeled with LysoTracker® Green. **h** Magnified view of the area enclosed by a red dashed square in (**g**)

A variety of cellular components (cargos) have been found to be delivered through TNTs [20]. Because the transfer of mitochondria through TNTs was thought to be a rescue of apoptotic cells [4], we wanted to understand if mitochondrion transportation occurs in macrophage CM-induced TNTs. Here we used the M0 CM as a representative treatment. Two groups of PANC-1 cells were sub-cultured and labeled with MitoTracker™ Green FM and MitoTracker™ Orange CMTMRos, separately. In Fig. 4a, we overlapped fluorescence and bright-field images to show the relative positions of the two mitochondria and the TNT connecting the two PANC-1 cells in the M0 CM. The time-lapse images in Fig. 4b–f show that two mitochondria moved head to head in a TNT, collided, and then moved together into the upper cell. (Please also see Video S1 in the Supplementary Information.) Such active transportation of mitochondria is thought to involve motor proteins [21]. Similar bidirectional transportation of vesicles containing carboxyl-modified quantum dots has been observed in TNTs formed in mouse tissues [22]. We also confirmed the co-localization of kinesin with the mitochondria within the TNT (Fig. S4 in the Supplementary Information). Mitochondria are believed to move along microtubules with the driving force from motor proteins such as kinesin and dynein. Although kinesin and dynein move in opposite directions on microtubules [23], in a TNT connecting two cells a mitochondrion might also be driven by two kinesin molecules on microtubules originated from the two cells. Therefore, bi-directional transportation could be possible in a TNT connecting two cells even with only one type of driving motor protein.

In addition to mitochondria, lysosomes were also found in the TNT connecting two cancer cells, as shown in Fig. 4g and h. Wong et al. pointed out that lysosomes could regulate mitochondrial functions by membrane contacts [24]. We conjectured that the co-transportation of mitochondria and lysosomes through TNTs could also be one of the PANC-1 cells’ responses to the macrophage CMs.

## Discussion

Macrophages play complicated roles in different kinds of cancers by secreting various cytokines [6, 25, 26]. They may also be involved in the interactions between cancer cells and other types of cells, such as fibroblasts [27]. It was generally regarded that M1 macrophages tend to perform pro-inflammatory activities in the tumor microenvironment. On the other hand, M2 macrophages in the tumor microenvironment can support tumor progression and malignancy [6, 7]. However, many inflammatory cytokines, such as IL-1, IL-6, IL-12, TNF-α, secreted by M1 macrophages are also important mediators of EMT [28, 29], which could improve cancer-cell motility and invasiveness. Kuwada et al. reported that both M1 and M2 macrophages induced EMT in pancreatic cancer cells [30]. Obviously the relationship between cancer-cell EMT and M1/M2 macrophages remains to be elucidated by further studies.

The paracrine loops between cancer cells and macrophages have long been recognized as essential interactions in the tumor microenvironment, which could modulate the phenotypes and functions of both types of cells [25]. On the other hand, direct contact between macrophages (M2a) and cancer cells was found to cause the dispersion of cancer cell aggregates [31]. In the present work, in order to avoid both the paracrine loops and direct contacts between cancer cells and macrophages, we studied the effects of macrophage CMs on the human pancreatic cancer cell line PANC-1. We found that M0 and M1 CMs induced the formation of TNTs and increased the migration speeds of the cancer cells, while only the M1 CM enhanced the invasiveness of PANC-1 cells. These effects were correlated with raised expression of the EMT-relevant protein Zeb-1, as well as the translocation of ZO-1 and β-catenin from membranes to the cytoplasms in the PANC-1 cells. Connexin 43, which is related to the p-38 mediated cell migration, was also increased by M0 and M1 CMs. Patheja and Sahu reported that the CM from M0 macrophages derived from U937 monocytes also induced the formation of TNTs between human breast cancer cells MCF-7 [32]. Interestingly, the TNTs could connect the MCF-7 cells and cytoplasmic fragments called microplasts, and transported mitochondria and vesicles from the parent cell to the microplast.

In contrast, although the M2 CM increased the migration speed of PANC-1 cells, it did not induce identifiable variations in TNTs, Zeb-1, and connexin 43. We thus suspected that, the THP-1 cell-derived M2 macrophages might not resemble those polarized in the tumor microenvironment, or the macrophages co-cultured with cancer cells [33]. Shiratori et al. have compared the behaviors of macrophages derived from THP-1 cells and human peripheral blood mononuclear cells (PBMCs). They suggested that THP-1 cell-derived macrophages are more suitable for studies involving phagocytosis and M1 polarization but not M2 polarization [34]. Our observation seemed to be consistent with this suggestion.

We employed FIB-SEM to reveal the cytoskeletons and tunnel-like structures embedded in the crowded space in a TNT. Sartori-Rupp et al. reported individual tunneling nanotubes (iTNTs) bundled in a TNT between neuronal cells by using cryo-transmission electron microscopy [13]. These iTNTs could serve as transportation channels for vesicles and mitochondria. We suspected that mitochondria or lysosomes might be squeezed through the TNT by the contraction force generated from actin–myosin interactions and the thrust force from co-localized motor proteins such as kinesin.

Macrophages have been proposed as novel cell-based carriers for nanomedicines because of their internalization capability and tumor- and hypoxia-approaching tendency [35]. The nanoparticle-carrying M1 macrophages were demonstrated to be able to migrate through the endothelial barrier into tissues surrounding a tumor [36]. TNTs were found to be formed between macrophages and breast cancer cells [11] or ovarian cancer cells [37]. The delivery efficacy of anti-cancer drug doxorubicin carried by macrophages could be enhanced by the TNTs between macrophages and tumor cells [37]. Therefore, manipulating cancer cells to produce TNTs in a controllable manner may pave a new way in cancer therapy.

## Conclusion

In summary, we have shown that M0 and M1 CMs induced the formation of TNTs in PANC-1 pancreatic cancer cells. The formation of TNTs was correlated with higher cell motility and EMT of the PANC-1 cells. We also observed bidirectional transportation of mitochondria in the TNTs. The findings may pave a way for the development of macrophage-targeting cancer therapy. However, in this paper we focused on the inner structures and transportation functions of the TNTs. Future experimental works using macrophages derived from monocytes isolated from peripheral blood and more pancreatic cancer cell lines will be necessary for validating if the macrophages can really enhance TNT formation in the tumor microenvironment.

## Methods

### Cell culture

The human pancreatic cancer cell line PANC-1 (ATCC CRL-1469) was obtained from the Bioresource Collection and Research Center (Hsinchu, Taiwan). We cultured PANC-1 cells in Dulbecco’s Modified Eagle Medium (DMEM; Gibco®, Thermo Fisher Scientific, Waltham, MA, USA) supplemented with 10% fetal bovine serum (FBS) and 1% antibiotic pen-strep-ampho. The human monocyte cell line THP-1 (ATCC TIB-202) was also obtained from the Bioresource Collection and Research Center. We cultured the THP-1 cells in RPMI supplemented with 10% FBS and 1% antibiotic pen-strep-ampho.

### Preparation of macrophage-conditioned media

In order to collect the macrophage CMs, we harvested 6 × 10^6^ THP-1 cells into 10 mL DMEM with 0.32 μM phorbol 12-myristate 13-acetate (PMA; Sigma-Aldrich, St. Louis, MO, USA). After treatment with PMA for 24 h, most of the monocytes attached onto the bottom of the culture dish and were considered to be differentiated into M0 macrophages [7, 30, 38, 39]. Further incubating the M0 macrophages with 10 pg/mL LPS + 20 ng/mL IFN-γ for 24 h or 20 ng/mL IL-4 + 20 ng/mL IL-13 for 72 h, we polarized the M0 macrophages into M1 and M2 macrophages, respectively [40]. The expression levels of relevant markers of M1 and M2 macrophages are shown in Fig. S1 in the Supplementary Information. We replaced the culture medium of each macrophage mentioned above with fresh DMEM, and conducted another 24 h of cultivation. The media were then collected and used as the macrophage CMs in the experiments.

### TNT quantification

We seeded PANC-1 cells at 1 × 10^5^ cells/mL in a μ-Dish (35 mm, high Grid-500; ibidi GmbH, Gräfelfing, Germany) under normal cultivation conditions (5% CO_2_ at 37 °C) overnight. Then the cells were cultured in the macrophage-CM for another 48 h. The cells were washed with phosphate-buffered saline (PBS, pH = 7.4) three times and then fixed with 3.7% formalin for 15 min at room temperature. We removed the fixative and washed the cells with PBS three times, and then used 1 μg/mL DAPI (Thermo Fisher Scientific) to stain the nuclei. After 10 min of incubation, the fixed cells were thoroughly washed with PBS and then imaged with a 20× phase-contrast objective installed on an inverted microscope (ECLIPSE Ti, Nikon, Tokyo, Japan). We counted the TNTs following these two criteria: (1) the tube connected two cells, and (2) the tube length was longer than 50 μm.

### FIB-SEM imaging

PANC-1 cells were cultured on well-cleaned and sterilized cover slips (22 mm × 22 mm). After treatment with the macrophage CMs, the cells were rinsed with PBS and then fixed with 3.7% formaldehyde for 15 min and later post-fixed with 1% OsO_4_ in PBS for 2 h. After triple PBS flushes, we replaced the medium with ethanol gradually from 10% (v/v in H_2_O) to 99.9%. The final samples were dried with a critical point dryer (EM CPD300, Leica Microsystems, Wetzlar, Germany). Before the FIB-SEM operation, the sample surface was sputtered with 10 nm gold to raise the conductivity. FIB-SEM operations were conducted with the FEI Helios NanoLab 660 (Thermo Fisher Scientific). The target area was firstly deposited with a 500 nm platinum protection layer via ion beam-induced deposition, rough-milled with 40 pA and then fine-milled with 7.7 pA gallium ion beams at a 30 kV acceleration voltage. All the SEM images were taken right after the FIB manipulations, acquired with a 25–50 pA beam current at a 1–5 kV acceleration voltage and an “in-lens” detector for the secondary electrons.

### Cell migration speed analysis

We seeded PANC-1 cells at 5 × 10^4^ cells/mL in a 35 mm dish under the normal cultivation condition overnight. The culture medium was then changed to the macrophage-CMs. We used a 10× phase-contrast objective to capture the time-lapse images of cells every 10 min for 48 h. In each experiment, the migration speeds of 45 cells were calculated using ImageJ.

### Cell invasion assay

A sample of ~ 3 × 10^5^ PANC-1 cells was re-suspended in a 6-well plate with the normal cultivation condition overnight. The culture media were changed to the macrophage CMs for another 48 hour culture. Then cells were starved for 24 hours in the serum-free medium prior to the invasion assay. About 5 × 10^4^ cells were seeded into a well of the Transwell chamber (ab235697, Basement Membrane, 8 μm, Abcam) containing the normal culture medium in the bottom chamber. Cells were cultured for 24 hours then labeled with the fluorescence dye (Ex. 530 nm/ Em. 590 nm) for 60 mins according to the kit’s instruction. The fluorescence intensities of invading cells were measured by using a plate reader (Synergy 2, BioTek Instruments, Winooski, VT, USA) to evaluate the cell invasion percentage.

### Quantitative polymerase chain reaction (qPCR)

Total RNA was purified using an RNeasy mini kit (217,604, Qiagen, Hilden, Germany) and reverse-transcribed to cDNA with the High-Capacity RNA-to-cDNA™ Kit (4,387,406, Applied Biosystems™, Thermo Fisher Scientific). qPCR was performed with PowerTrack™ SYBR Green Master Mix (A46110, Applied Biosystems™) and LightCycler 480 (Roche, Basel, Switzerland). The results were normalized with actin as internal control and evaluated by using the 2^−ΔΔCt^ method. Primers used in this study were synthesized by Genomics (New Taipei City, Taiwan) and listed in Table 1.

**Table 1:** Sequences of the primers for real-time PCR analysis

Gene	Primer sequence (5′ → 3′)
*VIM*	forward: AATGACCGCTTCGCCAACT reverse: ATCTTATTCTGCTGCTCCAGGAA
*ZEB1*	forward: TGAGCAAAACCATGATCCTAATGT reverse: CAGGTGCCTCAGGAAAAATGA
*M-Sec*	forward: TGCTCCAGAACCTGCATGAGGA reverse: AACTCAGGCAGCCTCGTGTCTA
*Cx43*	forward: GACAAGGTTCAAGCCTACTCAACTG reverse: TGTCCCCAGCAGCAGGAT
*ZO-1*	forward: CACGCAGTTACGAGCAAG reverse: TGAAGGTATCAGCGGAGG
*CTNNB1*	forward: AGCCGACACCAAGAAGCAGAGATG reverse: CGGCGCTGGGTATCCTGATGT
*IL1B*	forward: ATGATGGCTTATTACAGTGGCAA reverse: GTCGGAGATTCGTAGCTGGA
*TLR2*	forward: GCTCGGAGTTCTCCCAGTTTC reverse: GAGCTGCCCTTGCAGATAC
*CCL22*	forward: ATTACGTCCGTTACCGTCTG reverse: TAGGCTCTTCATTGGCTCAG
*ACTB*	forward: TGACGGGGTCACCCACACTGTGCCCATCTA reverse: CTAGAAGCATTTGCGGTGGACGATGGAGGG

### Confocal fluorescence imaging

For imaging the junction proteins, the PANC-1 cells were washed with PBS for three times and then fixed with anhydrous methanol for 10 min. The cells were permeabilized with 0.1% Triton X-100 for 10 minutes and blocked with 1% BSA in 1× PBS for 1 h at room temperature. Cells were then incubated with the antibodies of ZO-1 (MA3-39100-A488, 1:100, Thermo Fischer Scientific) and β-catenin (ab32572, 1:250, Abcam) overnight at 4 °C, and followed by a further incubation at room temperature for 1 h with the Goat Anti-Rabbit (A11008, 1:1000, Invitrogen, Alexa Fluor®488) secondary antibody. Nuclear DNA was labeled with DAPI (62,248, 1:1000, Thermo Fisher Scientific). Images were acquired with a confocal microscope (SP5, Leica Microsystems).

### Statistical analysis

All the data are presented as mean ± standard deviation. Comparisons between two groups were made by using a two-tailed Student’s *t*-test. Comparisons among data of more than two groups were conducted with one-way analysis of variance (ANOVA). Then the differences between specific two groups were checked with post hoc Tukey’s test.

## Supplementary Information

**Additional file 1: Video S1.** Bidirectional transportation of mitochondria in a TNT connecting two PANC-1 cells. Time intervals are 10 mins.

**Additional file 2: Fig. S1.** The mRNA levels of markers for M1 macrophage (a) IL-1β and (b) TLR-2, and (c) the marker for M2 macrophage CCL22, measured in the macrophages after the differentiation according to the protocols described in the Materials and Methods section. The data are from three independent experiments. ***, *P* < 0.005; *, *P* < 0.05 in comparison with those in THP-1 cells (post hoc Tukey’s test). **Fig. S2.** The ELISA results of EGF in (a) the conditioned media (CMs) of the THP-1 cells and macrophages, and (b) the CM of PANC-1 cells cultured in the macrophage CMs for 48 hours. The data are from three independent experiments. (c) The calibration curve of the optical density (OD) vs. the EGF concentrations. From this calibration curve, we learned that the EGF concentrations in panels (a) and (b) are all below the detection limit of ELISA. The ELISA kit was DY 236, DuoSet ELISA (R&D Systems, Minneapolis, MN, USA). The absorbance of the analytes were measured with a plate reader (Synergy 2, BioTek Instruments). **Fig. S3.** Formation of TNTs between two PANC-1 cells originally in contact (indicated by an arrow in the image at 0 min) in the M0 CM. This process is consistent with the “cell dislodgement” TNT formation mechanism. **Fig. S4.** Co-localization of kinesin (red) with the mitochondria (green) within a TNT. Most of the bright mitochondria were co-localized with the kinesin signal. The mitochondria were fused with green fluorescence protein in a stable cloned PANC-1 cell line. The kinesin was labeled with rabbit antibody (ab5629, abcam) then probed with DyLight 650-conjugated secondary antibody (ab96886, abcam).

## Data Availability

All data generated or analyzed during this study are included in this published article and its Additional files.

## Abbreviations

VIM: Vimentin
ZEB1: Zinc Finger E-Box Binding Homeobox 1
M-Sec: TNFα-inducible protein 2
Cx43: Connexin 43
ZO-1: Zonula occluden-1
CTNNB1: Catenin Beta 1
IL1B: Interleukin 1 Beta
TLR2: Toll Like Receptor 2
CCL22: C-C Chemokine type 22
ACTB: Actin Beta
TNTs: Tunneling nanotubes
CMs: Conditioned media
FIB-SEM: Focused ion beam-scanning electron microscope
EMT: Epithelial–mesenchymal transition
EGF: Epidermal growth factor
