# Supplementary material for: Effects of the media conditioned by various macrophage subtypes derived from THP-1 cells on tunneling nanotube formation in pancreatic cancer cells
Source: BMC Mol Cell Biol. 2022 Jul 6;23:26. doi: 10.1186/s12860-022-00428-3 (PMC9258106; doi:10.1186/s12860-022-00428-3)
Supplement: Supplementary file 2 — Additional file 2: Fig. S1. The mRNA levels of markers for M1 macrophage (a) IL-1β and (b) TLR-2, and (c) the marker for M2 macrophage CCL22, measured in the macrophages after the differentiation according to the protocols described in the Materials and Methods section. The data are from three independent experiments. ***, P < 0.005; *, P < 0.05 in comparison with those in THP-1 cells (post hoc Tukey’s test). Fig. S2. The ELISA results of EGF in (a) the conditioned media (CMs) of the THP-1 cells and macrophages, and (b) the CM of PANC-1 cells cultured in the macrophage CMs for 48 hours. The data are from three independent experiments. (c) The calibration curve of the optical density (OD) vs. the EGF concentrations. From this calibration curve, we learned that the EGF concentrations in panels (a) and (b) are all below the detection limit of ELISA. The ELISA kit was DY 236, DuoSet ELISA (R&D Systems, Minneapolis, MN, USA). The absorbance of the analytes were measured with a plate reader (Synergy 2, BioTek Instruments). Fig. S3. Formation of TNTs between two PANC-1 cells originally in contact (indicated by an arrow in the image at 0 min) in the M0 CM. This process is consistent with the “cell dislodgement” TNT formation mechanism. Fig. S4. Co-localization of kinesin (red) with the mitochondria (green) within a TNT. Most of the bright mitochondria were co-localized with the kinesin signal. The mitochondria were fused with green fluorescence protein in a stable cloned PANC-1 cell line. The kinesin was labeled with rabbit antibody (ab5629, abcam) then probed with DyLight 650-conjugated secondary antibody (ab96886, abcam). [file 12860_2022_428_MOESM2_ESM.docx]

**Effects of the Media Conditioned by Various Macrophage Subtypes Derived from THP-1 Cells on Tunneling Nanotube Formation in Pancreatic Cancer Cells**

Chia-Wei Lee^1^, Chia-Chen Kuo^1^, Chi-Jung Liang^2^, Huei-Jyuan Pan^1^,

Chia-Ning Shen^2,3,*^ and Chau-Hwang Lee^1,4,*^

^*^ Correspondence: cnshen@gate.sinica.edu.tw; clee@gate.sinica.edu.tw

^1^Research Center for Applied Sciences, Academia Sinica, Taipei 11529, Taiwan

^2^Genomics Research Center, Academia Sinica, Taipei 11529, Taiwan

^3^Department of Biotechnology and Laboratory Science in Medicine, National Yang Ming Chiao Tung University, Taipei 11221, Taiwan

^4^Institute of Biophotonics, National Yang Ming Chiao Tung University, Taipei 11221, Taiwan

**Supplementary Figures**


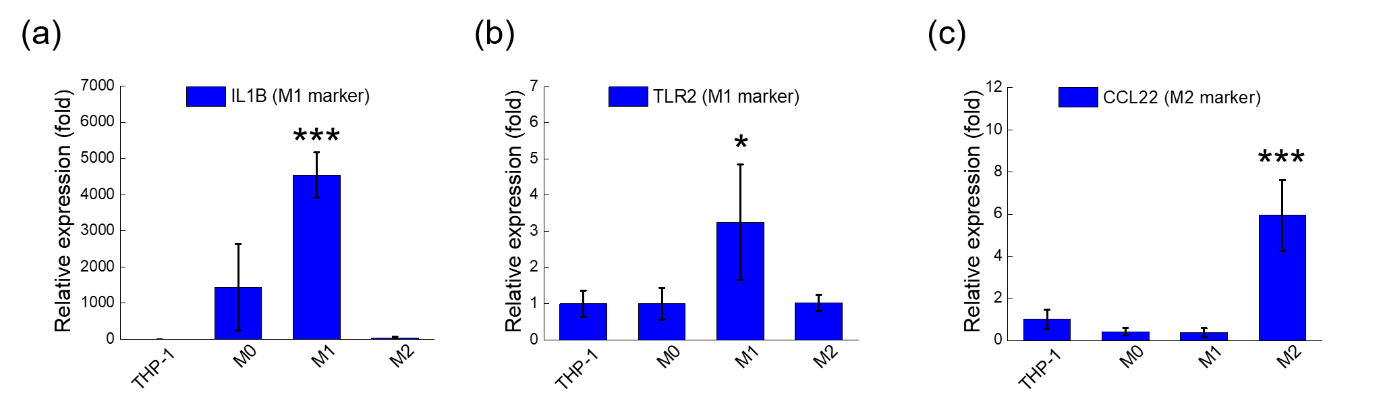


**Fig. S1.** The mRNA levels of markers for M1 macrophage (a) IL-1β and (b) TLR-2, and (c) the marker for M2 macrophage CCL22, measured in the macrophages after the differentiation according to the protocols described in the Materials and Methods section. The data are from three independent experiments. ***, *P* < 0.005; *, *P* < 0.05 in comparison with those in THP-1 cells (post hoc Tukey’s test).


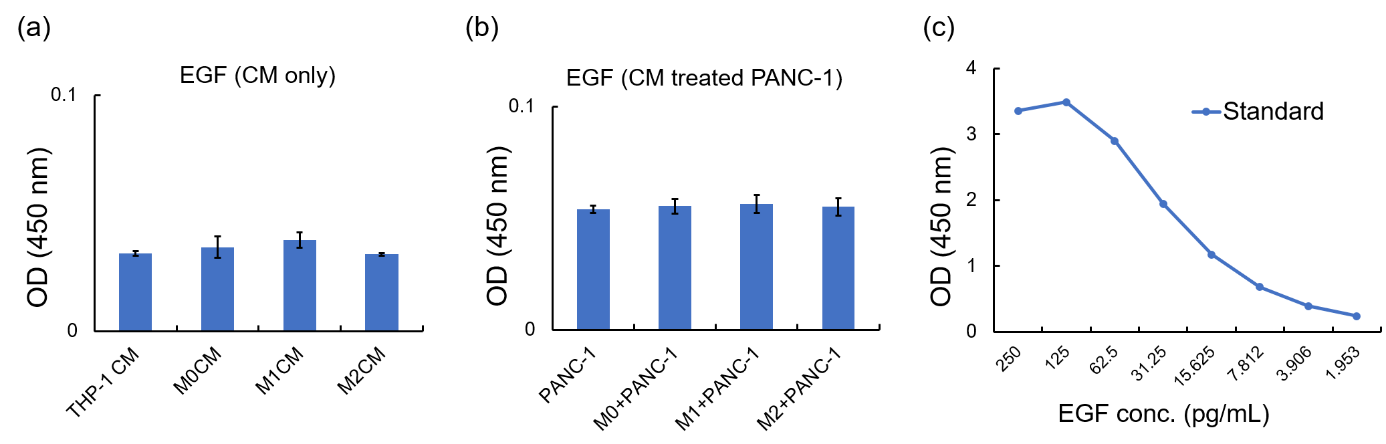


**Fig. S2.** The ELISA results of EGF in (a) the conditioned media (CMs) of the THP-1 cells and macrophages, and (b) the CM of PANC-1 cells cultured in the macrophage CMs for 48 hours. The data are from three independent experiments. (c) The calibration curve of the optical density (OD) vs. the EGF concentrations. From this calibration curve, we learned that the EGF concentrations in panels (a) and (b) are all below the detection limit of ELISA. The ELISA kit was DY 236, DuoSet ELISA (R&D Systems, Minneapolis, MN, USA). The absorbance of the analytes were measured with a plate reader (Synergy 2, BioTek Instruments).


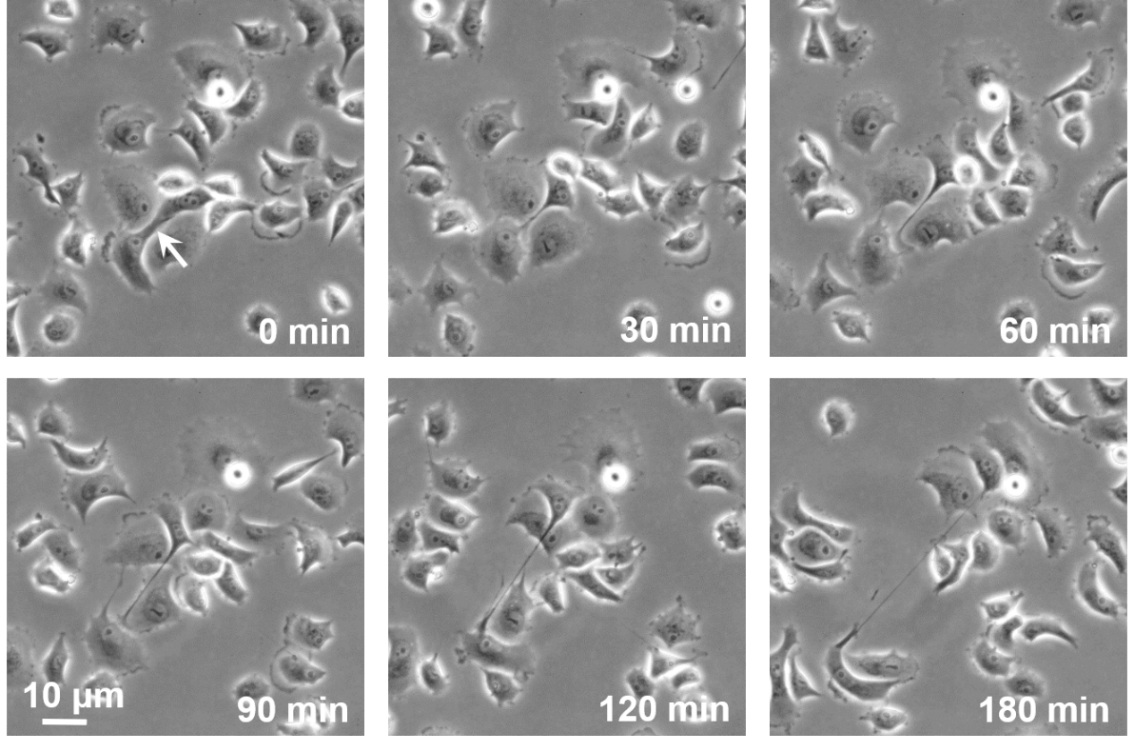


**Fig. S3.** Formation of TNTs between two PANC-1 cells originally in contact (indicated by an arrow in the image at 0 min) in the M0 CM. This process is consistent with the “cell dislodgement” TNT formation mechanism.


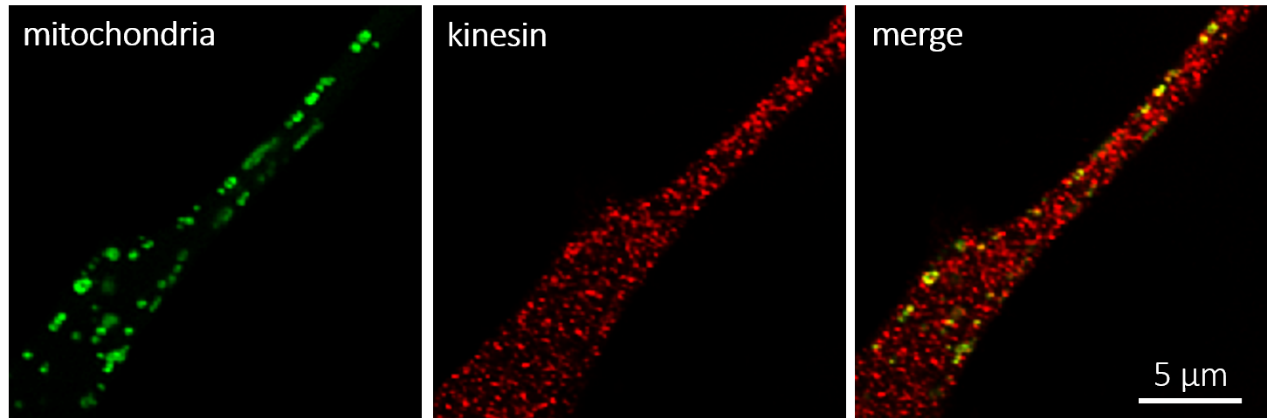


**Fig. S4.** Co-localization of kinesin (red) with the mitochondria (green) within a TNT. Most of the bright mitochondria were co-localized with the kinesin signal. The mitochondria were fused with green fluorescence protein in a stable cloned PANC-1 cell line. The kinesin was labeled with rabbit antibody (ab5629, abcam) then probed with DyLight 650-conjugated secondary antibody (ab96886, abcam).
